# Comparative physiology and transcriptome analysis allows for identification of lncRNAs imparting tolerance to drought stress in autotetraploid cassava

**DOI:** 10.1186/s12864-019-5895-7

**Published:** 2019-06-21

**Authors:** Liang Xiao, Xiao-Hong Shang, Sheng Cao, Xiang-Yu Xie, Wen-Dan Zeng, Liu-Ying Lu, Song-Bi Chen, Hua-Bing Yan

**Affiliations:** 1 Cash Crops Research Institute Guangxi Academy of Agricultural Sciences, Nanning, Guangxi 530007 People’s Republic of China; 2 Guangxi Crop Genetic Improvement and Biotechnology Key Lab, Nanning, Guangxi 530007 People’s Republic of China; 3 Tropic Crops Genetic Resources Institute, Chinese Academy of Tropical Agricultural Sciences, Danzhou, Hainan 571737 People’s Republic of China

**Keywords:** Autotetraploid, Cassava, Comparative transcriptomics, Drought stress, lncRNAs, Stomatal density

## Abstract

**Background:**

Polyploidization, pervasive among higher plant species, enhances adaptation to water deficit, but the physiological and molecular advantages need to be investigated widely. Long non-coding RNAs (lncRNAs) are involved in drought tolerance in various crops.

**Results:**

Herein, we demonstrate that tetraploidy potentiates tolerance to drought stress in cassava (*Manihot esculenta* Crantz). Autotetraploidy reduces transpiration by lesser extent increasing of stomatal density, smaller stomatal aperture size, or greater stomatal closure, and reducing accumulation of H_2_O_2_ under drought stress. Transcriptome analysis of autotetraploid samples revealed down-regulation of genes involved in photosynthesis under drought stress, and less down-regulation of subtilisin-like proteases involved in increasing stomatal density. UDP-glucosyltransferases were increased more or reduced less in dehydrated leaves of autotetraploids compared with controls. Strand-specific RNA-seq data (validated by quantitative real time PCR) identified 2372 lncRNAs, and 86 autotetraploid-specific lncRNAs were differentially expressed in stressed leaves. The co-expressed network analysis indicated that LNC_001148 and LNC_000160 in autotetraploid dehydrated leaves regulated six genes encoding subtilisin-like protease above mentioned, thereby result in increasing the stomatal density to a lesser extent in autotetraploid cassava. Trans-regulatory network analysis suggested that autotetraploid-specific differentially expressed lncRNAs were associated with galactose metabolism, pentose phosphate pathway and brassinosteroid biosynthesis, etc.

**Conclusion:**

Tetraploidy potentiates tolerance to drought stress in cassava, and LNC_001148 and LNC_000160 mediate drought tolerance by regulating stomatal density in autotetraploid cassava.

**Electronic supplementary material:**

The online version of this article (10.1186/s12864-019-5895-7) contains supplementary material, which is available to authorized users.

## Background

Cassava (*Manihot esculenta* Grantz) is a diploid plant (2n = 2× = 36) and an important cash and energy crop cultivated in Asia, Africa, and Latin America for its storage roots, making it critical for food security and economic development [1]. Water scarcity harms production when cassava is cultivated in severely water-deficit regions, and although cassava can tolerant a wide range of adverse environmental conditions including drought, this can limit growth and survival [2].

Drought stress increases oxidative damage in plants [3] and reduces photosynthesis [4, 5]. To cope with this, plants have evolved complex mechanisms such as deeper and thicker root systems [6], stomatal modulation systems including reduced aperture size and/or density to reduce water loss from transpiration [7, 8], and accumulation of osmotic adjustment compounds [9]. At low to moderate concentrations, reactive oxygen species (ROS) such H_2_O_2_ may act as second messengers in stress signalling. However, excessive H_2_O_2_ production can trigger progressive oxidative damage to plant cells. Antioxidant enzymes scavenge excess H_2_O_2_ and other ROS to protect plant cells from damage [10, 11].

Doubling of the whole genome to generate polyploidy is ubiquitous among higher plant species, and the change to a polyploidy state increases abiotic stress tolerance in crop species. For example, tetraploidy in *cenchrus*, *Arabidopsis* and *paulownia* improves drought tolerance by lowering H_2_O_2_ accumulation and enhancing ROS clearance [12–14]. Lower stomatal conductance and the abscisic acid (ABA) signalling pathway are involved in drought tolerance in the leaves of autotetraploid Rangpur lime (*Citrus limonia*) [15]. Autopolyploidization increases the potassium content and promotes tolerance to salinity stress in *Arabidopsis* [16]. Altered anatomy induced by polyploidy may confer stronger drought tolerance upon autotetraploid cassava [17]. However, the physiological and molecular advantages underlying these adaptations have not been widely investigated.

Transcriptome analyses have confirmed subtle changes due to autopolyploidy in *Arabidopsis* and *Paulownia tomentosa* × *Paulownia fortunei* under drought stress [13, 14]. Studies of long non-coding RNAs (lncRNAs), which are longer than 200 nucleotides in length and lack a coding sequence, have expanded our understanding of eukaryote transcriptome [18]. LncRNAs play important roles in many different biological processes in plants [19]. Thousands of lncRNAs associated with drought responses have been identified in several crop species due to the rapid development of omics sequencing technologies [20–24]. However, to date, only a limited number of lncRNAs, including *COOLAIR*, *COLDAIR*, *npc536*, *IPS1*, *LDMAR*, *PMS1T*, *DRIR*, *ELENA1* and *TL*, have been cloned and characterised [19, 25–31]. Overexpressing lncRNA *DRIR* can enhance drought tolerance in *Arabidopsis* [29].

In the current study, we characterised physiological differences between diploid progenitor (2×) and autotetraploid (4×) cassava under standard and drought conditions. Comparative transcriptome analysis was used to identify differentially expressed genes (DEGs) and lncRNAs in the leaves of 2× and 4× cassava under well-watered and dehydration conditions. Co-expression analysis revealed that two differentially expressed (DE) lncRNAs regulated six DEGs that improve drought tolerance in cassava by modulating stomatal density.

## Results

### Autotetraploid cassava displays stronger drought tolerance than diploid plants

**Fig. 1:**
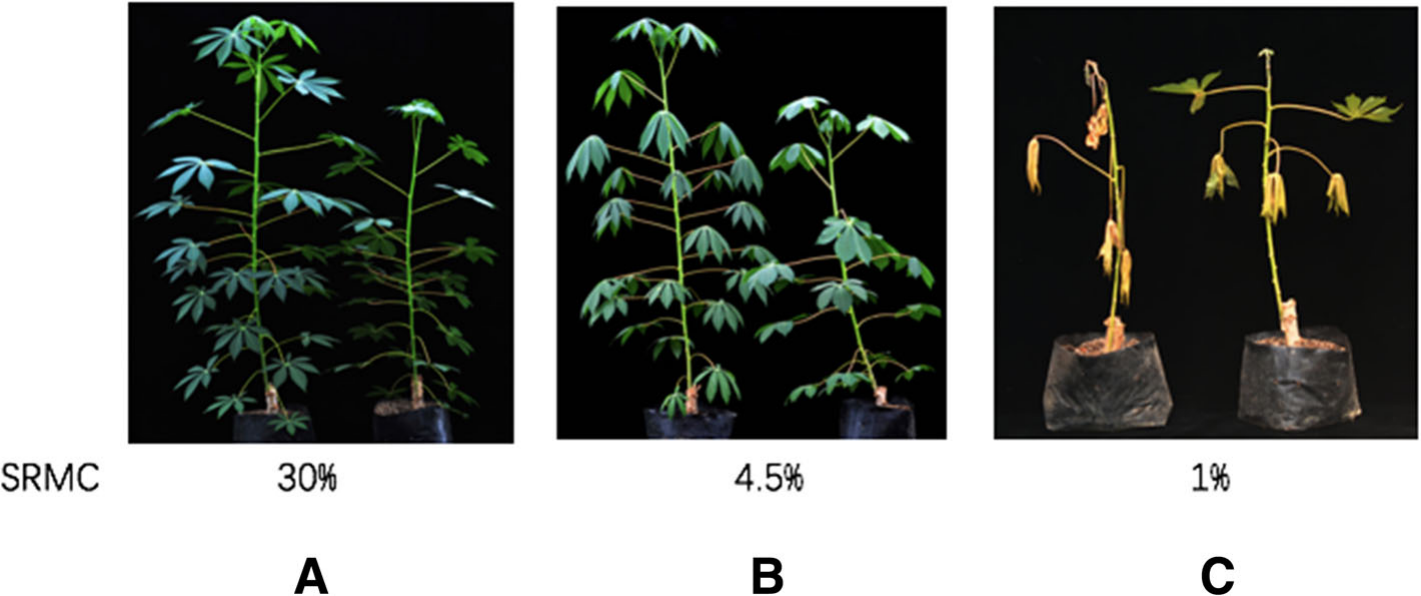
Phenotype changes in cassava plants in response to drought stress. On the left are 2× cassava ‘Xinxuan 048’ plants, and 4× plants are shown on the right. **a** Cassava plants (100-day-old) under normal conditions with 30% RSMC, **b** Plants after 15 days of water deprivation, with 4.5% RSMC, **c** Plants after water deprivation for 30 days (1% RSMC) and recovery for 2 days

**Fig. 2:**
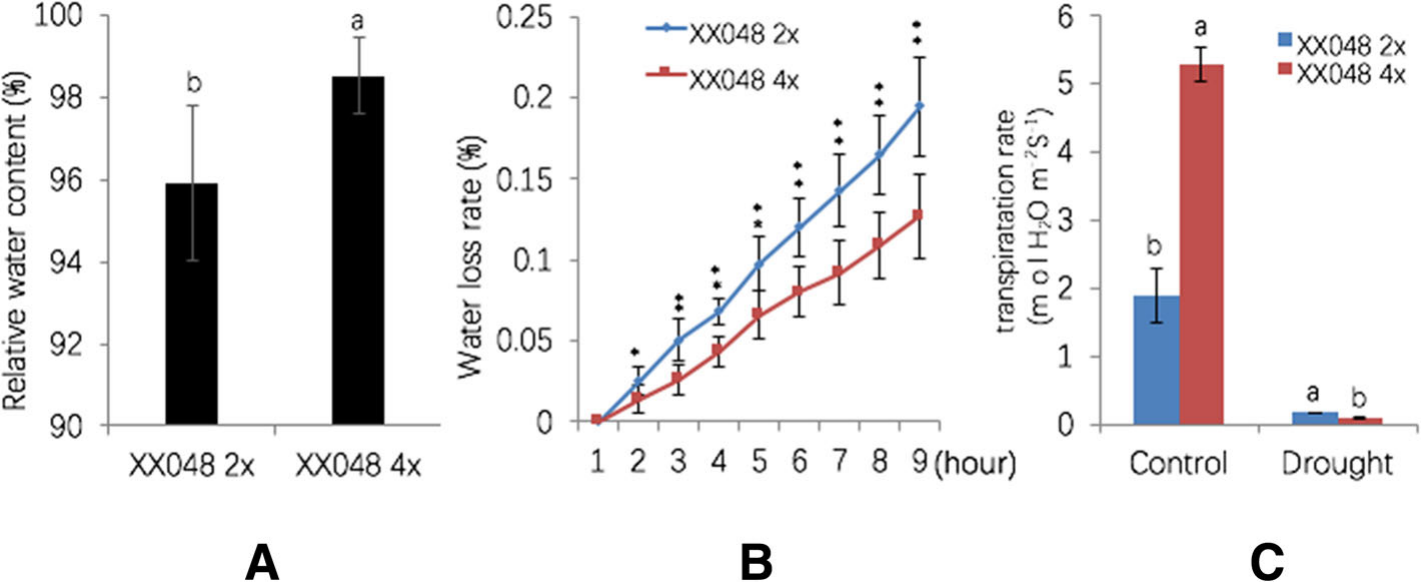
4× cassava plants enhanced drought tolerance by reducing transpiration. **a** Comparison of the RWC, **b** water losses percentage in detached leaves, **c** transpiration rate of seedlings in 3-month-old 2× and 4× cassava plants. Values are means ± SD (*p < 0.05, **p < 0.01, Student’s *t*-tests)

We exposed ‘Xinxuan 048’ 2× and 4× plants to soil with the same relative water content to compare their responses to drought stress. The RMSC was 30% for controls (Fig. 1a) and 4.5% for the drought stress treatment (Fig. 1b). After 15 days of withholding watering, at which time the RSMC was 4.5%, leaves of the 2× plants displayed moderate drooping, while leaves of 4× plants displayed only slight drooping (Fig. 1b). After withdrawing water for 30 days, followed by 2 days of recovery, all 2× plants were dead, while the growing points of 4× plants survived, and the upper leaves of autotetraploids remained green (Fig. 1c). Furthermore, the RWC of detached 4× leaves was higher than that of 2× plants (Fig. 2a). Analysis of water loss rate showed that 2× detached leaves lost water much more rapidly than those of 4× plants (Fig. 2b). Moreover, the transpiration rate of 4× seedlings was significantly lower than that of 2× seedlings (Fig. 2c). Thus, overall, 4× cassava plants were significantly more drought-tolerant than 2× plants.

### Autotetraploidy alters drought-mediated stomatal function and photosynthetic capacity

**Fig. 3:**
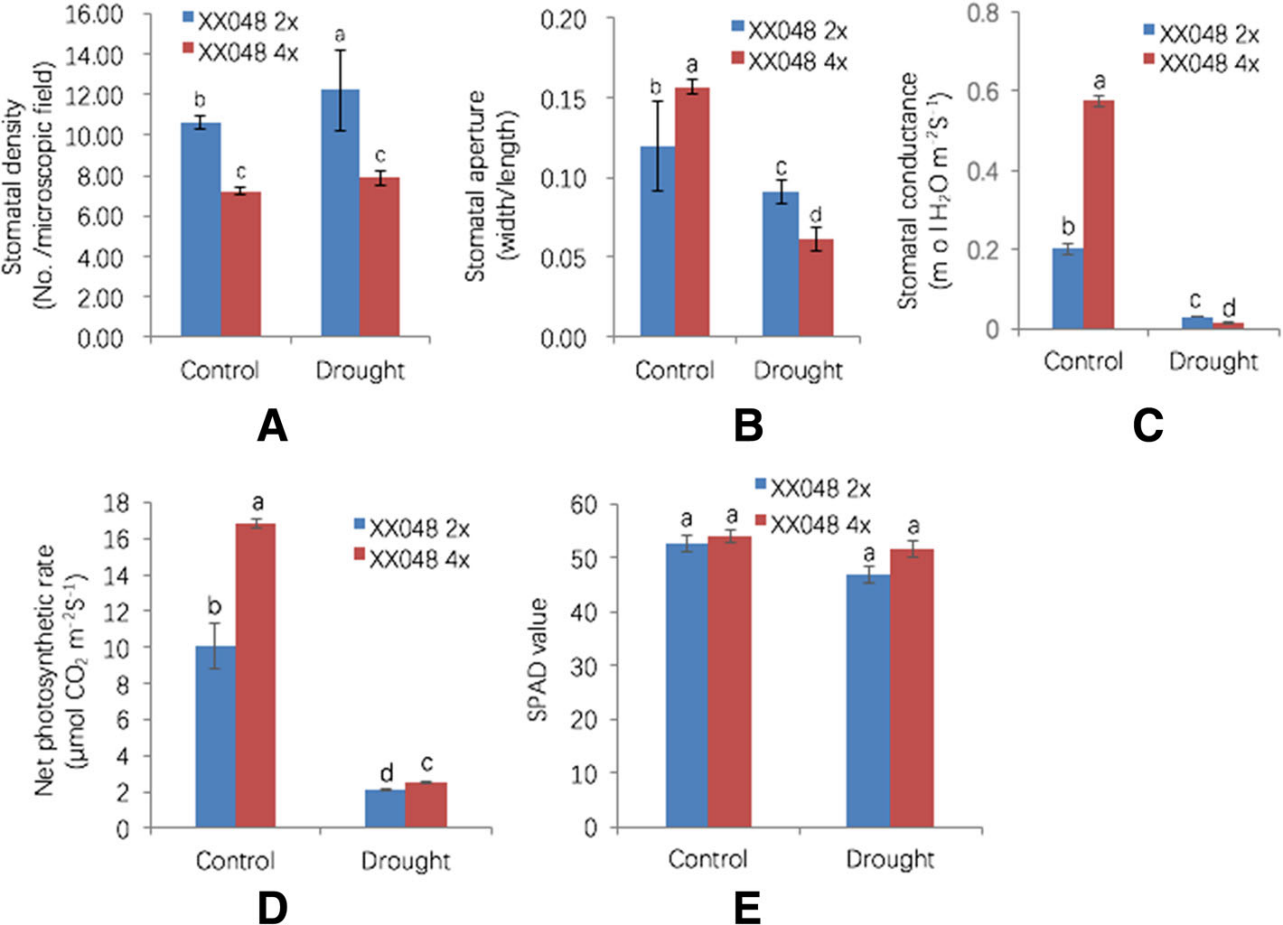
Changes in stomatal function and photosynthetic parameters induced by drought stress. **a** Stomatal density, **b** Stomatal aperture, **c** Stomatal conductance, **d** Net photosynthetic rate, **e** SPAD value. Values are means ± SD. Different letters represent significant differences at *p* < 0.05 (Duncan’s tests)

Since water loss occurs mainly through stomatal movement, the reduced water loss in 4× plants prompted us to investigate the stomatal function of the two genotypes under control and 4.5% RSMC conditions. The stomatal density of 4× plants (7.24) showed a significant reduction compared with that of 2× plants (10.64) under control conditions, and the density of both genotypes increased with increasing drought stress, but the density of 4× plants (7.88) remained significantly lower than that of 2× plants (12.24) in dehydrated leaves (Fig. 3a). The average stomatal aperture in 2× plants under control conditions was 0.12 μm, compared with 0.09 μm in dehydrated 2× plants. The average stomatal aperture in control 4× plants was ~ 0.16 μm, but this dropped 0.06 μm under drought condition (Fig. 3b). Thus, 4× leaves showed enhanced stomatal closure in response to drought, and stomatal conductance was markedly decreased in 4× leaves compared with 2× leaves under 4.5% RSMC (Fig. 3c). These results indicate that stress tolerance in 4× plants may be due to reduced transpiration rate via lesser extent increasing of stomatal density, smaller stomatal aperture size, and/or greater stomatal closure.

Next, we investigated photosynthesis parameters of the two genotypes. Photosynthesis is expected to be hindered due to lack of CO_2_ under drought stress. Although the stomatal conductance of 2× plants was greater than that of 4× plants under drought stress, the net photosynthetic rate of 4× plants was significantly higher (Fig. 3d). This result is consistent with the higher SPAD value of 4× plants, although the difference was not significant (Fig. 3e).

### Physiological effects of autotetraploidy

**Fig. 4:**
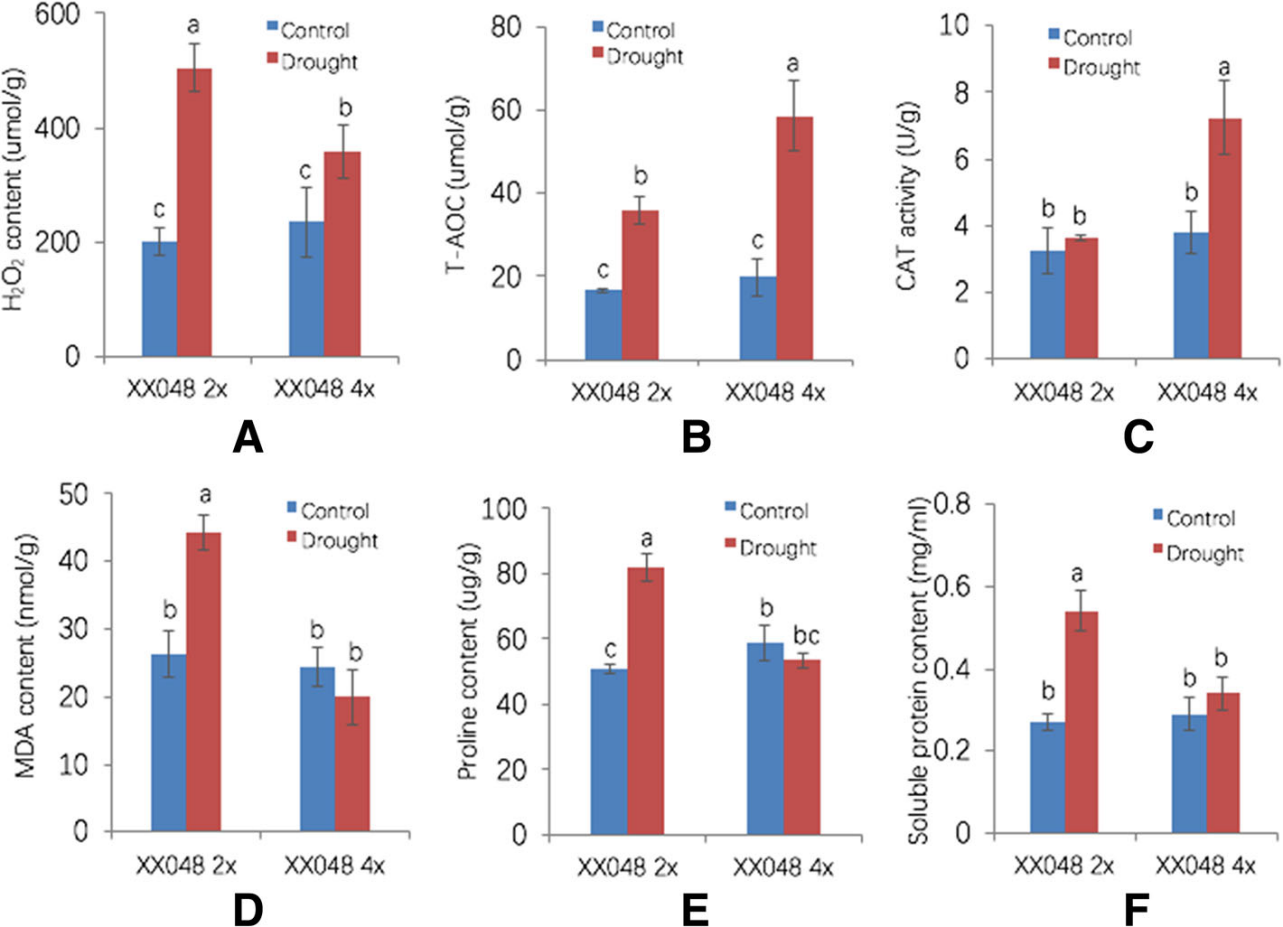
Changes in the physiological traits of 2× and 4× plants in response to drought stress. **a** H_2_O_2_ content, **b** T-AOC, **c** CAT activity, **d** MDA content, **e** Proline content, **f** Soluble protein content. Values are means ± SD. Different letters represent significant differences at *p* < 0.05 (Duncan’s tests)

Six physiological traits were used to investigate dynamic changes in response to drought stress in 2× and 4× plants. An increase of more than 150% in H_2_O_2_ production was observed in 2× leaves under drought stress, compared with less than 50% in drought-treated 4× leaves (Fig. 4a). By contrast, the relative increase in T-AOC and CAT activity following drought treatment was much less in 2× plants than 4× plants (Fig. 4b and c). The MDA content is an indicator of the degree of lipid peroxidation, which can reflect damage to plant cell membranes [32]. Compared with controls, the MDA content was dramatically elevated in 2× dehydrated leaves but decreased in 4× dehydrated leaves, although the reduced value is not significant (Fig. 4d). The proline and soluble sugar content were also increased significantly in 2× plants with increasing drought stress, but were relatively unchanged in 4× plants (Fig. 4e and f). These results indicate that osmolyte biosynthesis is not influenced by tetraploidy. Overall, tetraploidy resulted in reducing accumulation of H_2_O_2_, and hence the ability to alleviate cell membrane injury, thereby increasing drought tolerance in 4× plants.

### Identification of the lncRNAs and hierarchical clustering

**Fig. 5:**
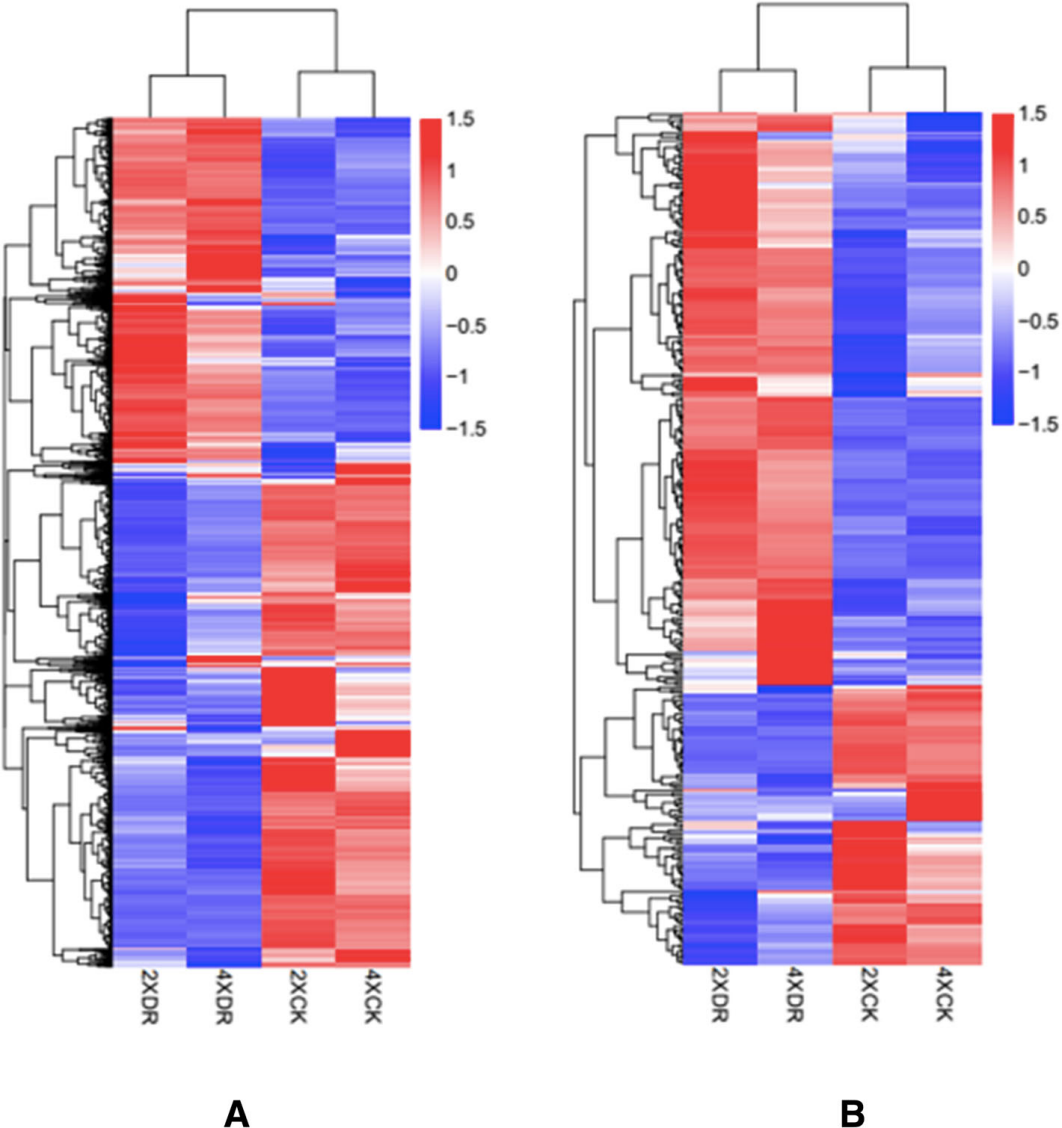
Hierarchical clustering of **a** mRNAs and **b** lncRNAs under control and drought stress conditions in 2× and 4× plants

In total, 1,232,088,086 bp raw reads were generated of 12 libraries by paired end sequencing. After moving the adapters and low-quality reads, 1,182,713,428 bp clean reads were mapped to the cassava genome. The mapped reads for each sample were assembled into transcripts using a reference-based approach. The correlation coefficient of the expression quantity of all transcriptome was more than 0.824 between each other among the 12 samples (Additional file 1: Figure S1). Expression levels of all coding genes and lncRNA transcripts were systematically estimated using FPKM values. In total, 2372 lncRNAs, including 821 antisense_lncRNAs and 1551 lincRNAs, were identified in this study (Additional file 2: Table S1). To calculate the degree of differential expression (DE) among lncRNAs, hierarchical clustering was performed using FPKM values of lncRNAs under control and drought stress conditions in 2× and 4× leaves. The results suggest that tetraploidization may have a limited effect on the mRNA transcriptome and lncRNA expression, since 2XCK and 4XCK clustered together, and 2XDR and 4XDR formed another cluster (Fig. 5a). Remarkably, significant DE was observed following drought stress in both diploid and autotetraploid cassava, suggesting that DE lncRNAs may play an important role in responses to drought stress (Fig. 5b).

### LncRNAs as potential miRNA precursors

By aligning miRNA precursors to the 2372 lncRNAs, we identified 18 lncRNAs as 21 known cassava miRNA precursors, including miR162, miR166c, miR408 and miR477c (Additional file 3: Table S2). A single lncRNA could serve as a precursor of several miRNAs, and different miRNAs could be targeted by the same lncRNA. Lnc_001314-miR171a was DE in response to drought.

### Comparison of mRNA transcripts under drought stress

In order to investigate transcriptional changes under water deficit conditions, we performed two pairwise transcriptomes comparisons; (2XDR_vs._2XCK) vs. (4XDR_ vs._ 4XCK), and (4XCK_vs._2XCK) vs. (4XDR_vs._2XDR).

**Fig. 6:**
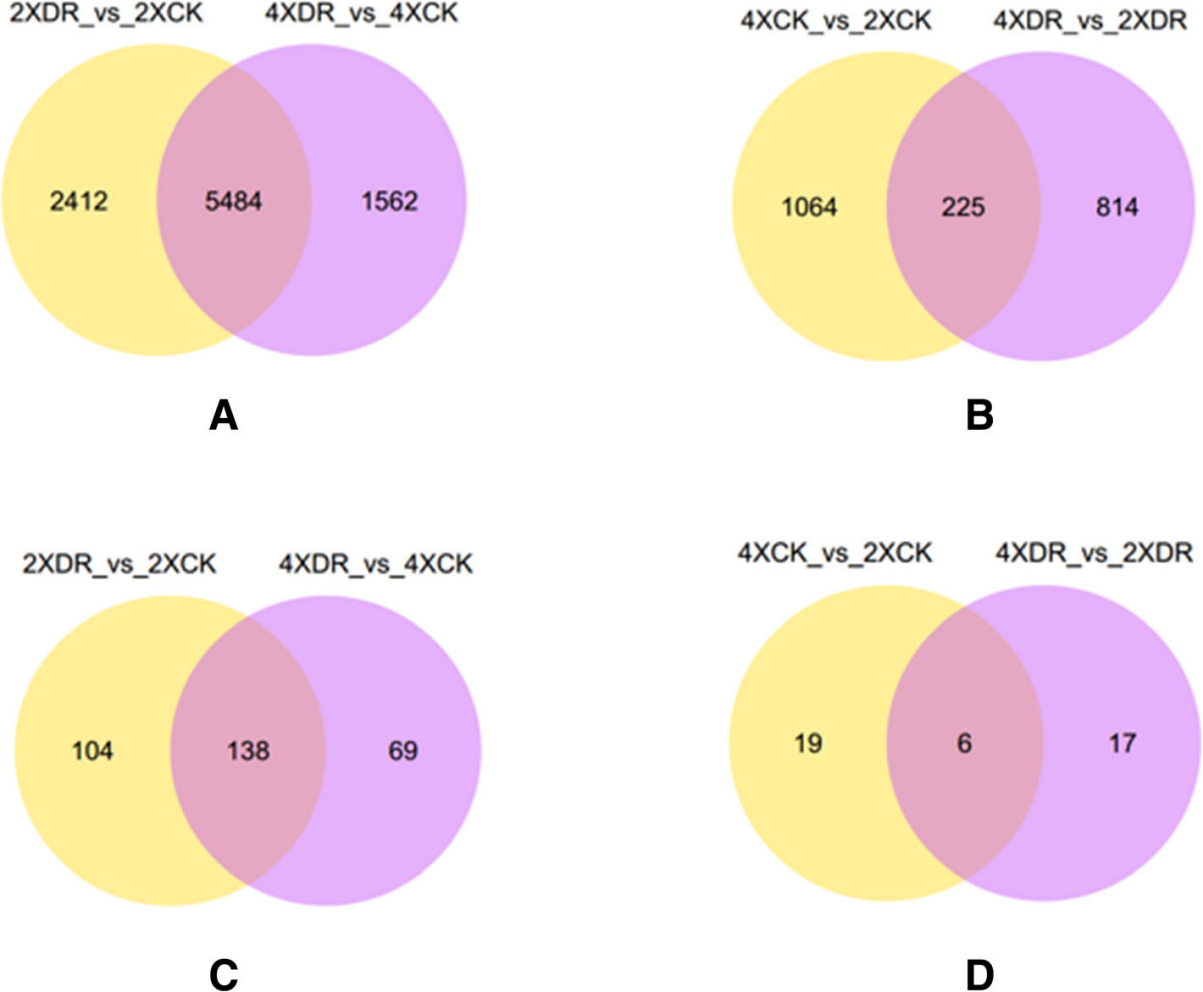
Venn diagrams showing the number of specific and common **a** DEGs and **c** DE lncRNAs in 2× and 4× plants in response to drought. The number of **b** DEGs and **d** DE lncRNAs in the comparison between 4× stressed leaves vs. 2× stressed leaves and 4× vs. 2× controls

Using a q-value < 0.05 and a fold change > 1 or < − 1 as thresholds, 1562 DEGs were found to be specifically responsive to drought in 4× sample (Fig. 6a), of which 687 genes were up-regulated and 875 were down-regulated (Additional file 4: Table S3). A total of 5484 genes were commonly expressed (Fig. 6a; Additional file 5: Table S4). We identified 2412 DEGs in 2× samples, including 1032 up-regulated and 1380 down-regulated genes (Fig. 6a; Additional file 6: Table S5). In 4× versus 2× plants subjected to dehydration, 814 DEGs were identified (Fig. 6b), including 288 down-regulated and 526 up-regulated DEGs (Additional file 7: Table S6). The reliability of the deep sequencing data was validated by quantitative real time PCR (qPCR) with gene-specific primers (Additional file 8: Table S7) for six randomly selected mRNAs (Additional file 9: Figure S2). The results showed that all the six genes displayed similar expression patterns in both RNA-seq and qPCR data. Notably, the number of drought-responsive mRNAs in 2× plants (2412) was higher than in 4× plants (1562), suggesting that 2× leaves might be more sensitive to drought than those of 4× plants, consistent with the more pronounced phenotype differences in 2× plants under water deficit stress.

A closer inspection of the 5484 common genes identified a number of down-regulated genes. In particular, almost all the genes encoding subtilisin-like proteases, except MANES_05G206800 and MANES_06G139800, were more strongly down-regulated in 2× leaves than in 4× leaves following dehydration (Table 1). We used the qPCR to detect the expression of seven genes in the four samples, the results showed that MANES_05G206800 and MANES_06G139800 turned out to be opposite trend with the RNA-seq data, while the other five were consistent (Additional file 10: Figure S3). The discrepancy between qPCR and RNA-seq could be attributed to different statistical method. Consistently, a subtilisin-like protease gene mutant, *sdd1–1*, exhibited a two- to four-fold increase in stomatal density in *Arabidopsis* [33]. *IiSDD1* was found to be autopolyploidy responsive and down-regulated by drought stress in autopolyploidy *Isatis indigotica* [34]. In the present work, the stomatal density was elevated in stressed 4× leaves (8.84%), but to a lesser extent than in 2× leaves (15.04%; Fig. 3a). Interestingly, the four subtilisin-like protease genes including, MANES_18G044300, MANES_18G039200, MANES_17G062600 and MANES_06G013200 were also found to be up-regulated expressed in 4× versus 2× plant subjected to dehydration (Additional file 7: Table S6). These results indicate a correlation between the regulation of stomatal density-related genes, resulting in a lesser extent of increasing stomatal density under dehydration stress, and consequently enhanced drought tolerance in 4× plants.

**Table 1:** Fold change in expression levels of subtilisin-like protease genes in 2× and 4× cassava plants under drought stress conditions

Gene ID	log2(fold_change) (2XDR/2XCK)	log2(fold_change) (4XDR/4XCK)	Gene_Description
MANES_16G094900	-1.80266	-1.41035	Subtilisin-like protease SBT1.7
MANES_12G067800	-2.62504	-2.61958	Subtilisin-like protease SBT5.3
MANES_16G104900	-2.97663	-2.28009	Subtilisin-like protease SBT5.3
MANES_01G158500	-2.52847	-1.79324	Subtilisin-like protease SBT5.3
MANES_14G157400	-1.25968	-0.952658	Subtilisin-like protease SBT2.5
MANES_18G039200	-3.19598	-1.76702	Subtilisin-like protease SBT1.7
MANES_04G146100	-1.50525	-1.21834	Subtilisin-like protease SBT1.7
MANES_17G062600	-3.20317	-2.16262	Subtilisin-like protease SBT5.3
MANES_05G175300	-3.99769	-3.34901	Subtilisin-like protease SBT1.6
MANES_03G066700	-1.53429	-1.1137	Subtilisin-like protease SBT2.5
MANES_06G013200	-2.2356	-0.571655	Subtilisin-like protease SBT1.7
MANES_01G267200	-2.32601	-1.57528	Subtilisin-like protease SBT5.3
MANES_05G041400	-5.43676	-3.05899	Subtilisin-like protease SBT1.1
MANES_11G013600	-1.35173	-0.546447	Subtilisin-like protease SBT1.6
MANES_06G020400	-1.56475	-1.38065	Subtilisin-like protease SBT2.5
MANES_18G044300	-4.17109	-3.55976	Subtilisin-like protease SBT1.7
MANES_06G139800	-5.6355	-5.65534	Subtilisin-like protease SBT1.7
MANES_05G206800	-3.01135	-3.03096	Subtilisin-like protease SBT5.3

Among the 5484 common genes that were down-regulated, genes involved in photosynthesis were particularly pronounced, including ferredoxin, ferredoxin-NADP reductase, beta-carbonic anhydrase, chlorophyll a-b binding protein, fructose-bisphosphate aldolase, photosynthetic NADH dehydrogenase subunit, and PsbP domain-containing protein 3. Expression of these genes was reduced in dehydrated 4× leaves more than in 2× leaves (Table 2), suggesting that photosynthesis was affected more in 4× plants than in 2× plants following drought treatment, consistent with the more pronounced reduction in stomatal aperture in 4× leaves under drought stress (Fig. 3b).

**Table 2:** Changes in differentially expressed genes involved in photosynthesis in 2× and 4× cassava leaves under drought stress conditions

Gene Id	log2(fold_change) (2XDR/2XCK)	log2(fold_change) (4XDR/4XCK)	Gene Description
MANES_07G020000	-3.06933	-3.23255	Ferredoxin
MANES_18G012800	-1.59929	-2.06096	Ferredoxin--NADP reductase
MANES_18G012800	-1.59929	-2.06096	Ferredoxin--NADP reductase
MANES_13G029900	-0.951361	-1.04625	Beta-carbonic anhydrase 4
MANES_15G183900	-0.728208	-0.870252	Chlorophyll a-b binding protein CP26
MANES_07G128500	-1.90678	-2.072	Chlorophyll a-b binding protein CP29.3
MANES_01G004600	-2.26458	-2.72572	Fructose-bisphosphate aldolase
MANES_08G083500	-0.783349	-1.10584	Fructose-1,6-bisphosphatase
MANES_12G007500	-0.863079	-1.63516	Photosynthetic NDH subunit of lumenal location 5
MANES_15G035500	-0.600976	-0.910963	Photosynthetic NDH subunit of subcomplex B 5
MANES_05G127800	-0.480897	-0.731987	PsbP domain-containing protein 3

Regulating the expression of transcription factors (TFs) is an efficient strategy for amplifying transcriptional responses. As shown in Table 3, members belonging to the WRKY, MYB, and ERF TF families were represented. In particular, all seven ERF family members were down-regulated in 4× plants under drought stress, and all 14 WRKY members except MANES_10G127100 and MANES_11G066500 were up-regulated under drought stress (Table 3). Consistently, these three families of TFs appear to play important roles in drought stress signalling [35–37].

**Table 3:** Members of three transcription factor families differentially expressed in 2× and 4× cassava plants under drought stress conditions

2XDR/2XCK		4XDR/4XCK	
Gene ID	log2(fold_change)	Gene ID	log2(fold_change)
ERF family		ERF family	
MANES_06G131100	-2.09415	MANES_10G056300	-7.56729
MANES_01G271300	-1.97542	MANES_03G056100	-7.43179
MANES_18G093800	-1.23836	MANES_14G029500	-4.15486
MANES_13G148300	-1.15324	MANES_01G085200	-2.55651
MANES_12G047500	-0.985827	MANES_02G042400	-1.99999
MANES_13G049600	0.853316	MANES_03G106300	-1.67408
MANES_14G039000	1.02722	MANES_06G131200	-1.43908
WRKY family		WRKY family	
MANES_14G018600	-5.2904	MANES_10G127100	-1.48452
MANES_04G062400	-0.990689	MANES_11G066500	-1.16416
MANES_16G134800	0.506777	MANES_05G008500	0.550684
MANES_05G203900	0.724048	MANES_05G106900	0.552488
MANES_18G098700	0.890046	MANES_05G004300	0.874532
MANES_13G068100	1.50057	MANES_01G007600	0.916557
MANES_02G189600	1.56809	MANES_01G235400	0.917217
MANES_03G008900	1.97703	MANES_10G002200	1.18082
MANES_01G047200	2.5873	MANES_03G206400	1.66717
MYB family		MANES_01G230000	1.80879
MANES_02G041300	-2.38862	MANES_07G142400	2.03957
MANES_03G052800	-1.46426	MANES_03G051300	3.2512
MANES_03G052800	-1.46426	MANES_09G123700	3.35004
MANES_08G095200	-1.44376	MANES_16G129000	4.94482
MANES_02G047900	-1.14407	MYB family	
MANES_05G098700	-1.11459	MANES_01G035100	-1.31543
MANES_03G077700	-1.10159	MANES_18G042000	-1.27147
MANES_01G035100	-1.05067	MANES_04G074900	0.599183
MANES_14G077700	-0.870616	MANES_03G098700	0.970182
MANES_05G007400	-0.821554	MANES_01G226200	0.996307
MANES_11G094800	-0.800502	MANES_03G098700	0.970182
MANES_14G071900	-0.794355		
MANES_01G194000	-0.626848		
MANES_05G177900	0.76616		
MANES_01G147500	1.31855		
MANES_05G114400	4.49487		

### Comparison of lncRNA transcripts under drought stress

Similarly, (2XDR_vs._2XCK) vs. (4XDR_ vs._ 4XCK) and (4XCK_vs._2XCK) vs. (4XDR_vs._2XDR) comparisons identified DE lncRNAs. Among the lncRNAs with a *q*-value <0.05 and a fold change > 1 or < − 1, 69 DE lncRNAs were specific to drought-stressed 4× leaves (Fig. 6c), including 45 up-regulated and 24 down-regulated DE lncRNAs (Additional file 11: Table S8). A total of 138 DE lncRNAs were identified in both genotypes (Fig. 6c; Additional file 12: Table S9), including 104 drought-responsive lncRNAs specific to 2× plants (Fig. 6c), of which 72 were up-regulated while 32 were down-regulated (Additional file 13: Table S10). A Venn diagram (Fig. 6d) showed among 17 DE lncRNAs, 11 were down-regulated and six were up-regulated in 4× stressed leaves vs. 2× stressed leaves (Additional file 14: Table S11). The sequences of DE lncRNAs are listed in Additional files 15 and 16: Tables S12 and S13.

**Fig. 7:**
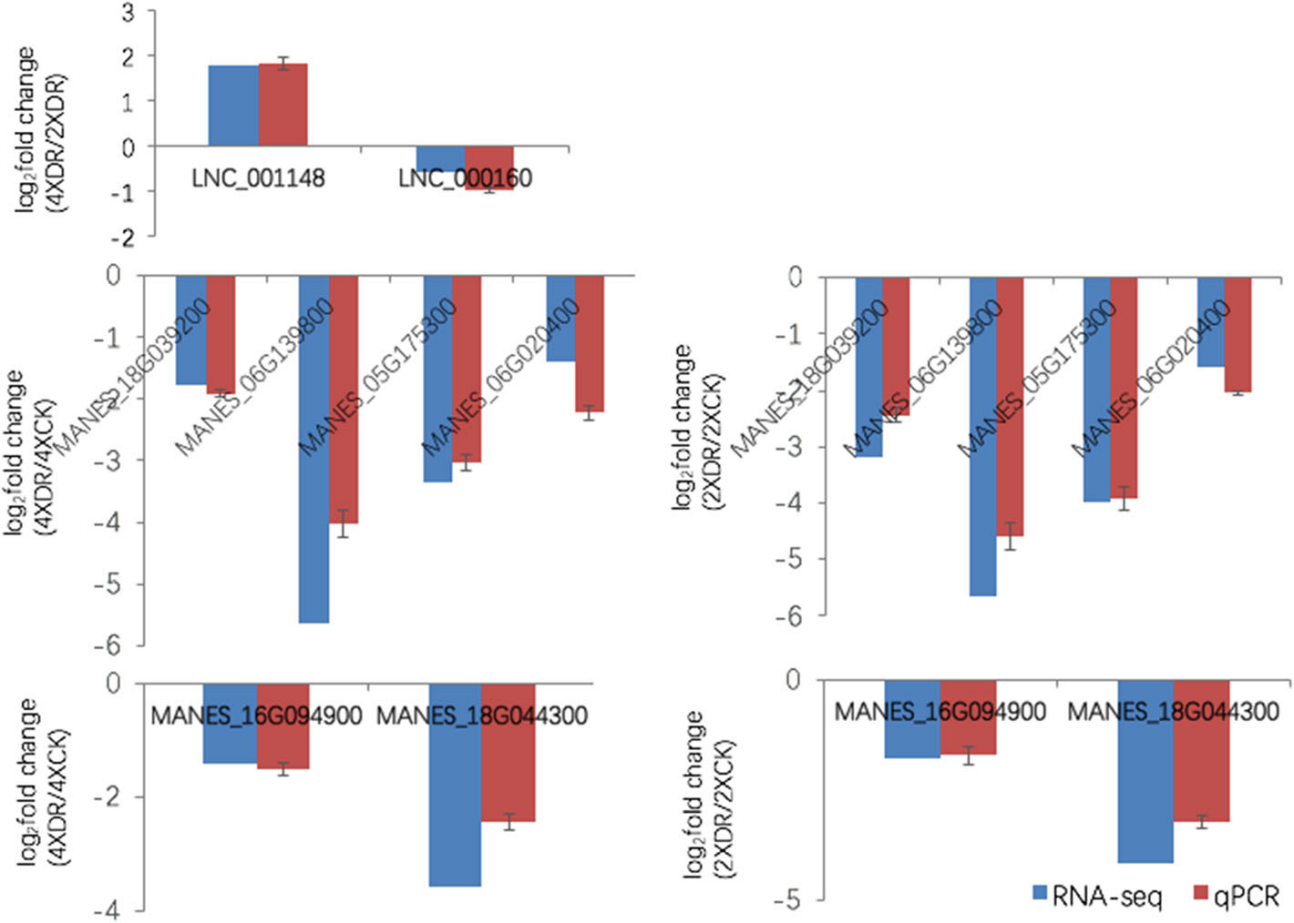
The qPCR validation of the expression of four lncRNAs with the co-expressed target genes between Me4XDR and Me4XCK. Data are means ± SD of three biological experiments. Cassava β-actin was used as an internal control

**Fig. 8:**
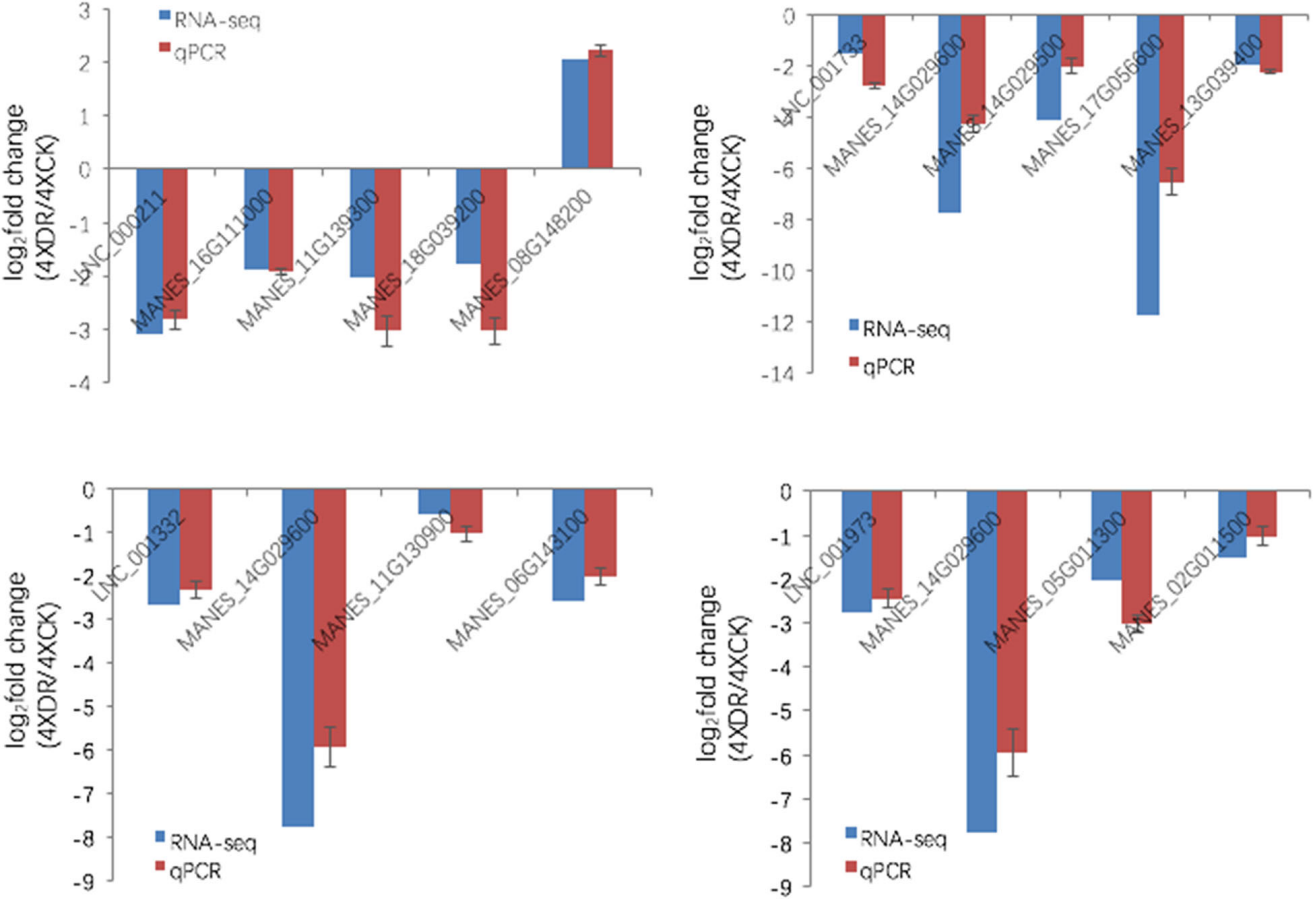
Comparison of expression results from RNA-Seq and qPCR methods for the two lncRNAs and co-expressed subtilisin-like protease family members. Data are means ± SD of three biological experiments. Cassava β-actin was used as an internal control

In order to explore the functions of the 86 lncRNAs specific to 4× plants, we constructed a co-expression network and identified target genes associated with drought tolerance in 4× vs. 2× plants under drought stress (Additional files 17 and 18: Tables S14 and S15). We used the qPCR to detect the expression of four lncRNAs with the corresponding trans target genes in the two samples, Me4XDR and Me4XCK. The results indicated that the expression is consistent with the RNA-seq result (Fig. 7). Remarkably, we found that two of the DE lncRNAs might regulate six subtilisin-like protease DEGs; LNC_001148 (lincRNA) may regulate MANES_18G039200 (SBT1.7), MANES_06G139800 (SBT1.7), MANES_05G175300 (SBT1.6) and MANES_06G020400 (SBT2.5), while LNC_000160 (antisense lncRNA) may target MANES_16G094900 (SBT1.7) and MANES_18G044300 (SBT1.7). To validate the putative relationship between the two DE lncRNAs and six DEGs, expression levels were examined by qPCR. The results revealed similar lncRNA expression patterns to those obtained in the RNA-seq (Fig. 8), which suggests that the two lncRNAs identified using deep sequencing may target genes encoding subtilisin-like proteases via co-expression.

The functions of most of the 86 DE lncRNAs remain largely unknown, therefore, the genes co-expressed with 86 DE lncRNAs overlapping with DEGs were subjected to functional enrichment analysis using KEGG (Additional file 19: Table S16). The results indicated that 20 pathways were found responsive to drought stress. They included galactose metabolism, pentose phosphate pathway, plant hormone signal transduction, glycolysis/gluconeogenesis, biosynthesis of secondary metabolites and brassinosteroid biosynthesis, etc. (Additional file 20: Table S17).

## Discussion

Autopolyploidy confers advantages over diploid counterparts, often manifested in enhanced adaptation to adverse environmental conditions. However, the molecular advantages remain largely unknown. Herein, we demonstrated the importance of autotetraploidy in response to drought in cassava. The 4× cassava plants potentiated stronger antioxidant and photosynthesis capacities than 2× plants, helping them cope better with drought stress. Autotetraploidy elevated the expression levels of two lncRNAs co-expressed with genes encoding subtilisin-like proteases regulating stomatal density under drought stress. Additionally, autotetraploidy decreased the stomatal density on the leaf epidermis, which in turn improves the RWC and reduces water loss, enhancing drought tolerance in cassava plants.

ROS participate in signal transduction, but are also toxic to cell membranes when present in excessive quantities, and can affect plant drought susceptibility [38]. We observed that H_2_O_2_ in 4× stressed leaves were significantly lower than in 2× leaves (Fig. 4a). To detoxify excess drought-induced ROS, plants have developed a complex antioxidant system [39]. We found that 4× plants possessed a more efficient enzymatic antioxidant system involving CAT and T-AOC for reducing accumulation of H_2_O_2_ under drought stress (Fig. 4b and c). MANES_05G1307001, homolog of catalase isozyme 1 in *Ricinus communis*, is up regulated in both of 2× and 4× stressed leaves compared with normal condition. However, the fold change of MANES_05G1307001 in 4× is more than that in 2×. Therefore, autotetraploidy may be associated with the regulation of antioxidation ability. Some antioxidant enzymes were found to be up-regulated in stressed leaves, while others were down-regulated, suggesting cellular redox status may be complex and dynamic. We found that levels of UDP-glucosyltransferase were increased more or reduced less in leaves of 4× plants in response to drought compared with 2× leaves (Additional file 5: Table S4). Consistently, overexpression of *Arabidopsis* UGT79B2/B3 significantly enhances plant tolerance to drought stress by modulating anthocyanin accumulation, which enhances ROS scavenging [40]. Our results suggest that DEGs encoding UDP-glucosyltransferase may be tightly associated with improved drought tolerance in 4× by modulating ROS levels.

Stomata are surrounded by two guard cells in the epidermis that regulate the shape and size of the pore aperture [41]. One of the earliest adaptive responses to drought in cassava leaves is stomatal closure and/or decreased stomatal density to reduce water loss [42]. Stomata control the balance between the uptake of CO_2_ for photosynthesis and the release of water by modulating transpiration, thereby governing water use and abiotic stress tolerance [43]. It has been demonstrated that reducing the number of stomata does not affect carbon fixation due to increased CO_2_ concentration [44]. Therefore, the decreased photosynthetic capacity of 4× plants is consistent with a lower transpiration rate, which results, at least in part, from enhanced stomatal closure during drought treatment (Fig. 3b-d). It is known that photosynthesis-related genes are down-regulated after drought treatment in many plants [45]. However, we found that the net photosynthesis rate of 4× plants remained higher than that of 2× plants under dehydration conditions, which might be attributed to higher SPAD values in 4× plants (Fig. 3e). Thus, 4× plants may maintain a higher photosynthetic rate, facilitating better adaptation to drought stress conditions by meeting increased energy demand.

Our transcriptomic data indicates that autotetraploidy influences the expression of genes encoding TFs involved in drought stress in cassava, and general response mechanisms integrating hormone signalling in response to external stimuli. TFs are efficient amplifiers of transcriptomic responses that regulate differential gene expression attributed to autotetraploidy. ERFs are key regulatory hub proteins in hormone and regulatory ROS-responsive gene expression that confer abiotic stress tolerance [37]. Some WRKY and MYB TFs are components of ABA-mediated stomatal movement involved in drought responses [36, 46]. Three DE TF families were found to be in autotetraploid *Arabidopsis* under drought stress conditions [13].

A complex regulatory system controls drought tolerance in cassava. Most previous research in this area has focused on coding genes. LncRNAs are an important class of regulators in diverse biological processes involving complex mechanisms [47], and numerous lncRNAs have been identified in a few crop species including wheat (*Triticum aestivum*) [48], *Medicago truncatula* [49], *Brassica napus* [50], maize (*Zea mays*) [51] and cotton (*Gossypium spp.*) [52]. In our current study, we discovered 2372 lncRNAs, including 821 antisense_lncRNAs and 1551 lincRNAs, from 12 libraries. Li et al. [53] identified 682 lncRNAs from nine cassava samples. Differences between our current results and these previous results might be attributed to (i) differences in experimental design and transcriptome analysis, since the work of Li et al. [53] was based on 15-day-old seedling tissues under polyethylene glycol-simulated drought stress, and samples were collected from shoot apices and leaves; (ii) differences in bioinformatics strategies, since CPAT [54] and Pfam Scan [55, 56] were employed to filter transcripts with coding potential and screen candidate lncRNAs. LncRNAs could execute their functions to respond to stress in either cis-acting or trans-acting in the genome via diverse mechanisms in plant [57, 58]. Based on comparative transcriptome analysis, lncRNA16397 was found to be involved in *Phytophthora infestans* resistance by co-expression glutaredoxin in tomato (*Lycopersicon esculentum* Mill.) [59]. In the present study, LNC_001148 and LNC_000160, 898 bp and 2688 bp, respectively, were down-regulated by drought treatment in both 2× and 4× cassava plants. Co-expression analysis revealed that they appeared to regulate six genes encoding subtilisin-type proteinases. *SDD1*, belong to subtilisin serine proteinase family, which are known to negatively regulate stomatal density and distribution in *Arabidopsis* [33]. *IiSDD1* participates not only in the drought responsive pathways, but also involves in autopolyploidy *I. indigotica* evolution [34]. Thus, relative to 2× dehydrated leaves, the higher expression of LNC_001148 and/or lower expression of LNC_000160 in 4× leaves may result in less down-regulation of target genes encoding subtilisin-type proteinases, and hence lesser extent of increasing stomatal density, thereby enabling 4× plants to better adapt to drought stress. Exactly how the two lncRNAs confer drought tolerance in cassava will be studied in future work.

In Table S14, LNC_000211 was down regulated with fold change more than 8 times following drought treatment in 4× plant specifically. LNC_000211 could trans regulate MANES_16G111000 (encoding Dehydration-responsive element-binding protein), MANES_11G139300 (encoding ERF), MANES_18G039200 (encoding Subtilisin-like protease SBT1.7), MANES_08G148200 (encoding thioredoxin-like 1–1), respectively. Previous studies indicated that these four target genes were involved in drought tolerance in plants [33, 37, 60, 61]. Therefore, LNC_000211 may play an important role in mediating the tolerance to drought in 4× cassava plant. Based on the KEGG enrichment analyses, we found that the trans target genes of the 86 DE lncRNAs were involved in galactose metabolism, pentose phosphate pathway, brassinosteroid biosynthesis, etc. The three pathways were reported to be associated with drought responsive in sugarcane (*Saccharum officinarum* L.), purging nut (*Jatropha curcas*) and *Arabidopsis*, respectively [62–64]. Taken together, our results suggest that 4× cassava implements divergent mechanisms to modulate the response to drought stress.

The current understanding of lncRNA regulation in response to drought stress is in its infancy in 4× cassava. These findings provide a comprehensive view of 4× DE lncRNAs, which will enable in-depth functional analysis.

## Conclusion

Our study demonstrated that tetraploidy potentiates tolerance to drought stress in cassava. The co-expressed network analysis indicated that LNC_001148 and LNC_000160 in autotetraploid dehydrated leaves regulated six genes encoding subtilisin-like protease, thereby result in increasing the stomatal density to a lesser extent in autotetraploid cassava. This study helps to explain the role of autotetraploidy in conferring drought tolerance, and indicates the evolutionary potential of polyploidy in abiotic stress tolerance.

## Methods

### Water deficit treatment and water recovery

Cassava variety ‘Xinxuan 048’ (2× and 4×) used in this study were original from our lab [65]. Stem-propagated plants were grown in plastic pots (30 cm in height × 40 cm in diameter) containing well-mixed soil in March 2017. Each pot contained one cutting, and were placed in a greenhouse under a 16 h light/8 h dark photoperiod at the Guangxi Academy of Agricultural Sciences (GXAAS). Two-month-old cassava plants of each genotype were well-watered before drought stress treatment. Before dehydration treatment, each potted plant was watered until it was saturated to ensure consistency of water content. The moisture content of soil was measured by a AZS-100 soil moisture sensor (TRIME-PICO32, Germany). Control plants were well watered every 4 days. A relative soil moisture content (RSMC) of 30, 4.5% (after 15 days of withholding watering) and 1% (after 30 days of withholding watering until the top buds of diploid cassava displayed obvious wilting) was used for controls and drought stress treatments for two time points, respectively. After recovery for 2 days, the survival rate was determined and plants were photographed. All experiments were repeated in triplicate.

### Measurement of water loss rate

Seventy-day-old plants were used to calculate the water loss rate, for which the fourth, fifth and sixth leaves (counting from the top of the plant) and petioles were excised from each plant. Three plants were included for each genotype. Detached leaves were placed on filter paper in a culture room under a 16 h light/8 h dark photoperiod. The abaxial surface was placed facing up for dehydration, and leaves were weighed every hour. Water loss was estimated from the percentage of fresh weight lost relative to the initial fresh weight. All experiments were repeated in triplicate.

### Relative water content (RWC) assay

Four plants each of each genotype were used to measure the RWC. Seventy-day-old plants were detached at the fifth leaf (counting from the top of the plant) and weighed immediately (M1), then placed in water for 18 h under dark conditions, dried using a filter paper, then weighed again (M2). Saturated leaves were finally oven-dried for 24 h at 65 °C to a constant weight (M3), and the RWC was measured using eq. 1:

1 $$ \left(\mathrm{M}1\hbox{-} \mathrm{M}3\right)/\left(\mathrm{M}2\hbox{-} \mathrm{M}3\right)\times 100\% $$

### Drought stress treatment and plant sampling

One hundred-day-old cassava plants were used for drought stress analysis. Before dehydration treatment, each potted plant was watered until it was saturated to ensure consistency of water content. The moisture content of soil was measured by a AZS-100 soil moisture sensor (TRIME-PICO32, Germany). Control plants were well watered every 4 days. RSMC of 30 and 4.5% (after 15 days of withholding watering) was used for controls and drought stress treatments, respectively, and all treatments included three plants per genotype. The fifth leaf of each plant was used to measure stomatal aperture size, photosynthetic capacity, and physiological indices. The lower epidermis of leaves was used for examination as described previously by Zhou et al. [65]. The length and width of stomata were measured using 200 guard cells, and the number of stomatal openings was counted in 10 microscopic fields using three independent replicates. A LI-6400 portable photosynthesis system was used to measure the net photosynthetic rate, transpiration rate, and stomatal conductance. The relative chlorophyll content in the fifth leaves of each plant was determined by a soil plant analysis development (SPAD) value measured by an SPAD-502 Plus chlorophyll meter (Konica Minolta, Japan).

Physiological indices, namely catalase (CAT) activity, total antioxidant capacity (T-AOC), H_2_O_2_ content, and malondialdehyde (MDA) content, were estimated using reagent kit cat. # A007–2, A015, A064 and A003 (Institute of Nan Jing Jian Cheng Bioengineering), respectively. Proline content and total soluble protein content were measured using the method of Bates et al. [66] and Guy et al. [67], respectively.

Plants used for RNA-seq were treated as described above, and parallel leaves were frozen in liquid nitrogen and stored at − 80 °C.

### Library construction and deep sequencing

A total of 3 μg of RNA per sample was used as input material for RNA sample preparation. Whole-transcriptome library preparation and high-throughput sequencing were performed by Novogene Bioinformatics Technology Co., Ltd. (Beijing, PR China). Three replicates were generated for each control (2XCK and 4XCK) and treatment (2XDR and 4XDR). A total of 12 libraries were constructed, which included Me2XCK-1, Me2XCK-2, Me2XCK-3, Me2XDR-1, Me2XDR-2, Me2XDR-3, Me4XCK-1, Me4XCK-2, Me4XCK-3, Me4XDR-1, Me4XDR-2 and Me4XDR-3. All the libraries were sequenced on an Illumina Hiseq 2500 platform, and 125 bp paired-end reads were generated. Clean reads were obtained by removing reads containing adapters and poly-N sequences, and low-quality reads were also removed from raw data. Clean reads were aligned to the reference genome using TopHat v2 [68]. Both scripture (beta2) [69] and Cufflinks (v2.1.1) [70] were employed to assemble mapped reads for each sample into transcripts using a reference-based approach.

Coding potential was predicted using one or more of CNCI [71], CPC [72], Pfam-scan [73, 74] and PhyloCSF [75], and sequences without coding potential were considered candidate lncRNAs.

### Identification of DEGs and DE lncRNAs

Cuffdiff (v2.1.1) was used to calculate fragments per kilobase of transcript per million mapped reads (FPKM) values of both lncRNAs and coding genes in each sample [70]. Gene FPKMs were computed by summing the FPKMs of transcripts in each gene group. To assess the three biological replicates, the log_2_ (fold_change)-transformed FPKM values were performed. Cuffdiff provides statistical routines for determining DE in digital transcript using a model based on the negative binomial distribution [70]. A default q-value < 0.05 was set as the threshold for DE. Genes with log_2_ (fold_change) values > 0 were deemed up-regulated, while genes with log_2_ (fold_change) values < 0 were considered down-regulated.

### Trans target genes prediction of lncRNAs

Trans targets genes of lncRNAs were identified from expression correlations between lncRNAs and coding genes using custom scripts. The pearson correlation coefficient method was used to calculate the correlation between lncRNA and mRNA among samples. Correlations corresponding to a coefficient > 0.95 or < − 0.95 were selected for the functional enrichment analysis.

### KEGG enrichment analysis

We used KOBAS software to test the statistical enrichment of the genes co-expressed with DE lncRNAs overlapping with DEGs in KEGG pathways (http://www.genome.jp/kegg/) [76].

### Quantitative real time PCR validation

Total RNA was used to synthesise cDNA using a PrimeScript RT Reagent Kit (TaKaRa). Three technical replicates and three biological replicates were included for each experiment, and qPCR was performed using SYBR Premix Ex Taq [77].

## Additional files

Additional file 1: **Figure S1**. The pearson correlation coefficient of lncRNA between the 12 samples in this study. (TIF 19628 kb)

Additional file 2: **Table S1**. Expression levels of 2372 lncRNAs detected in this study. (XLSX 649 kb)

Additional file 3: **Table S2**. LncRNAs as miRNA precursors. (XLSX 10 kb)

Additional file 4: **Table S3**. The 1562 DEGs specific to 4× plants in response to drought. (XLSX 150 kb)

Additional file 5: **Table S4.** The 5484 DEGs common to both 2× and 4× plants in response to drought. (XLSX 467 kb)

Additional file 6: **Table S5**. The 2412 drought-responsive DEGs specific to 2× plants. (XLSX 244 kb)

Additional file 7: **Table S6**. The 814 DEGs in 4× stressed leaves vs. 2× stressed leaves. (XLSX 91 kb)

Additional file 8: **Table S7**. Sequences of qPCR primers used in this study. (XLSX 12 kb)

Additional file 9: **Figure S2**. Validation of the expression of six mRNAs selected randomly identified by RNA-seq using qPCR. Six mRNAs were selected randomly from 4XDR and 4XCK libraries. Bars represent means ± SD of three biological replicates. Cassava β-actin was used as an internal control. (TIF 11225 kb)

Additional file 10: **Figure S3**. Comparison of the expression of seven genes encoding subtilisin-like protease between Me2XDR_vs._Me2XCK and Me4XDR_vs._Me4XCK detected by qPCR. Bars represent means ± SD of three biological replicates. Cassava β-actin was used as an internal control. (TIF 16466 kb)

Additional file 11: **Table S8**. The 69 DE lncRNAs specific to 4× plants in response to drought stress. (XLSX 13 kb)

Additional file 12: **Table S9**. The 138 DE lncRNAs common to both 2× and 4× leaves in drought-stressed plants. (XLSX 17 kb)

Additional file 13: **Table S10**. The 104 DE lncRNAs specific to 2× plants in response to drought stress. (XLSX 15 kb)

Additional file 14: **Table S11**. The 17 DE lncRNAs in 4× vs. 2× plants subjected to drought stress. (XLSX 9 kb)

Additional file 15: **Table S12**. Sequences of the 69 DE lncRNAs specific to 4× plants in response to drought stress. (XLSX 51 kb)

Additional file 16: **Table S13**. Sequences of the 17 DE lncRNAs in 4× vs. 2× stressed leaves. (XLSX 21 kb)

Additional file 17: **Table S14**. A list of trans target genes of the 69 DE lncRNAs specific to 4× plants in response to drought stress, and Pearson correlations between lncRNAs and their corresponding target genes. (XLSX 411 kb)

Additional file 18: **Table S15**. A list of trans target genes of the 17 DE lncRNAs in 4× vs. 2× stressed leaves, and Pearson correlations between lncRNAs and their corresponding target genes. (XLSX 193 kb)

Additional file 19: **Table S16**. The transcript IDs co-expressed with 86 DE lncRNAs overlapping with DEGs in 4× leaves compared with 2× leaves under drought stress. (XLSX 47 kb)

Additional file 20: **Table S17**. The top 20 KEGG enrichment pathways of the genes co-expressed with 86 DE lncRNAs overlapping with DEGs in 4× leaves compared with 2× leaves under drought stress. (XLSX 50 kb)

## Data Availability

Not applicable.

## Abbreviations

ABA: abscisic acid
CAT: catalase
DE: differentially expressed / differential expression
DEG: differential expressed gene
LncRNA: long non-coding RNA
MDA: malondialdehyde
ROS: reactive oxygen species
RSMC: relative soil moisture content
RWC: relative water content
SD: standard deviation
SPAD: soil plant analysis development
TF: transcriptional factor
