# Supplementary figures and images for: Comparative physiology and transcriptome analysis allows for identification of lncRNAs imparting tolerance to drought stress in autotetraploid cassava
Source: BMC Genomics. 2019 Jun 21;20:514. doi: 10.1186/s12864-019-5895-7 (PMC6588902; doi:10.1186/s12864-019-5895-7)

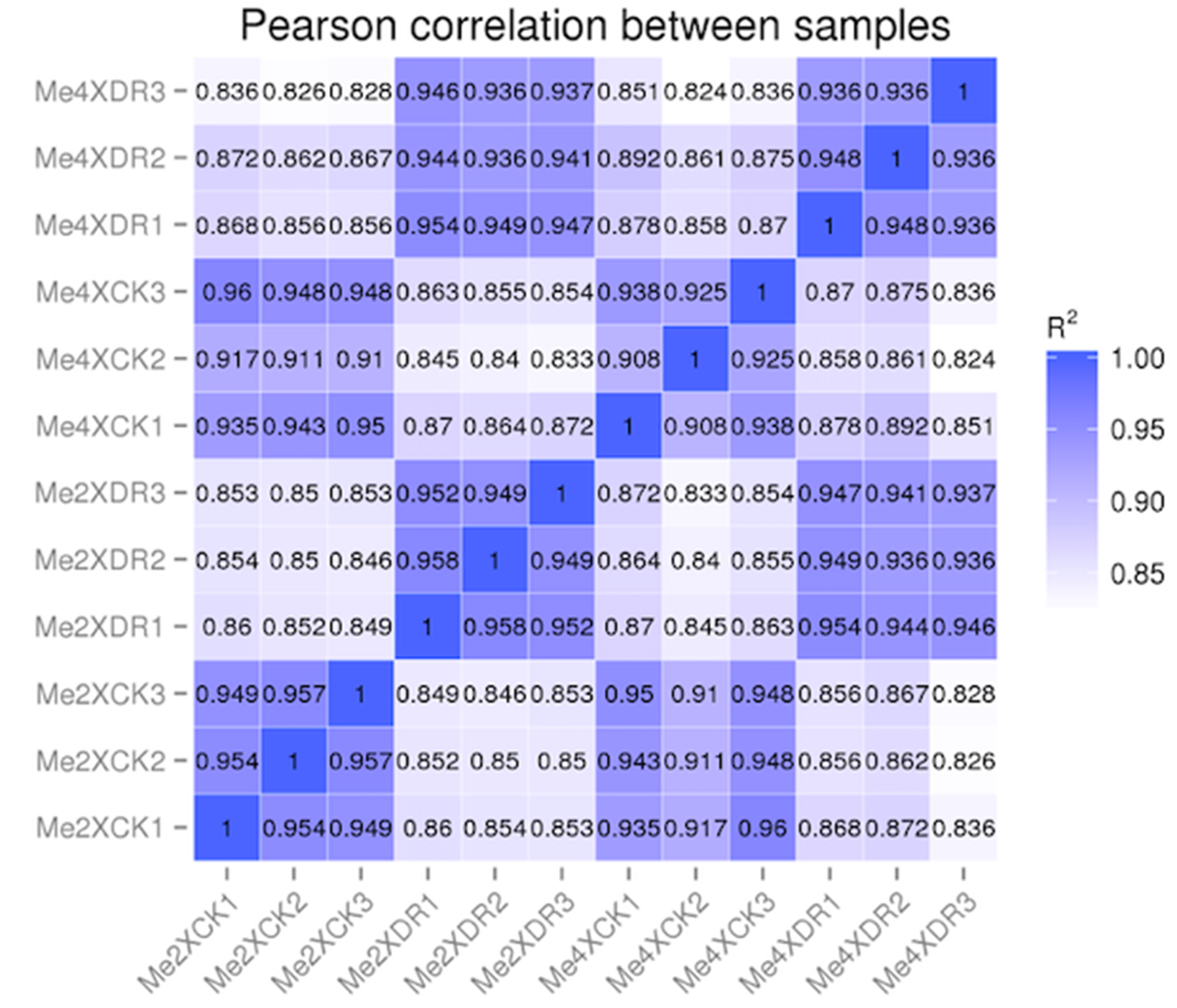

Supplement: Supplementary file 1 — Figure S1. The pearson correlation coefficient of lncRNA between the 12 samples in this study. (TIF 19628 kb) [file 12864_2019_5895_MOESM1_ESM.tif]

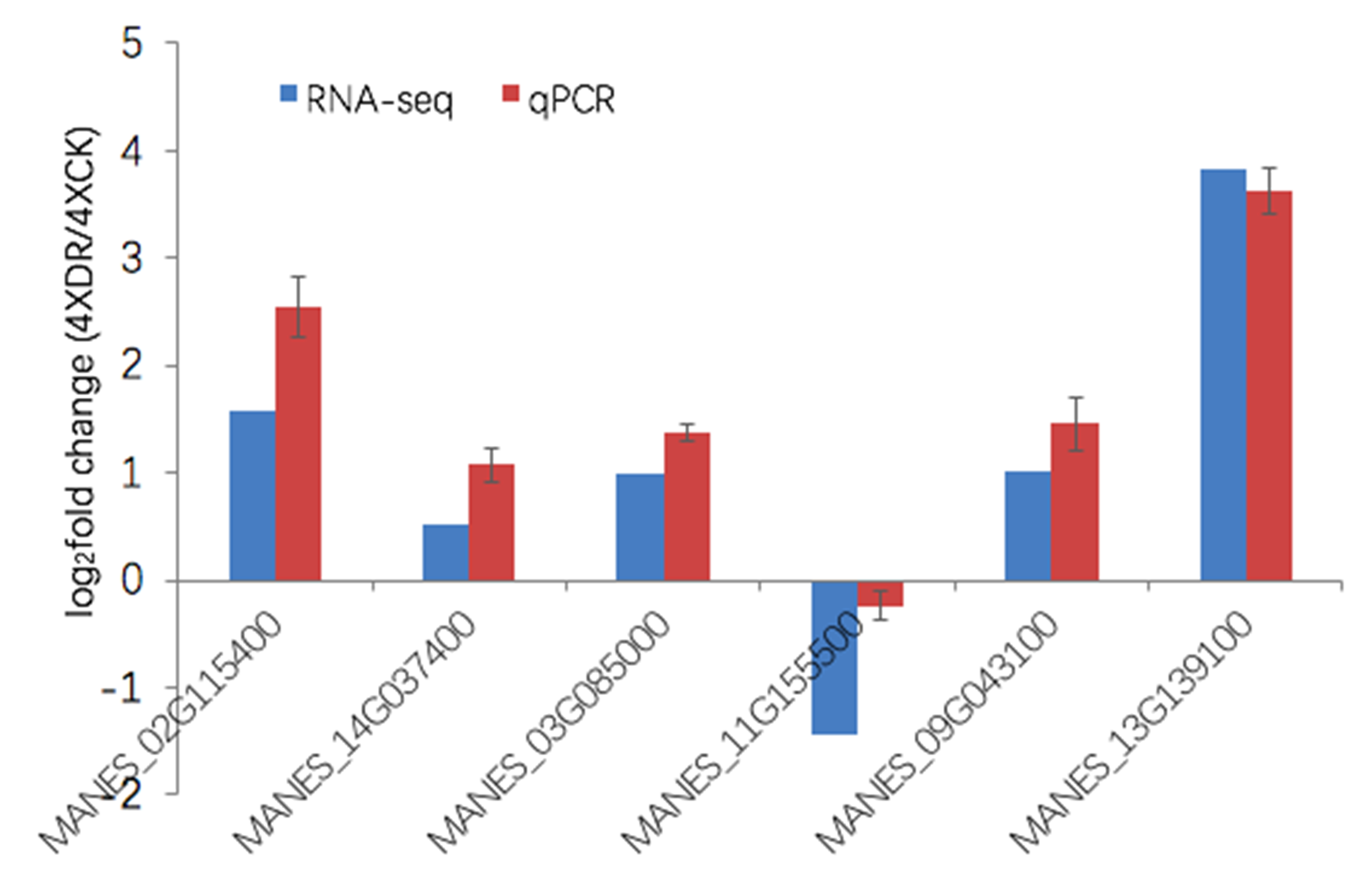

Supplement: Supplementary file 9 — Figure S2. Validation of the expression of six mRNAs selected randomly identified by RNA-seq using qPCR. Six mRNAs were selected randomly from 4XDR and 4XCK libraries. Bars represent means ± SD of three biological replicates. Cassava β-actin was used as an internal control. (TIF 11225 kb) [file 12864_2019_5895_MOESM9_ESM.tif]

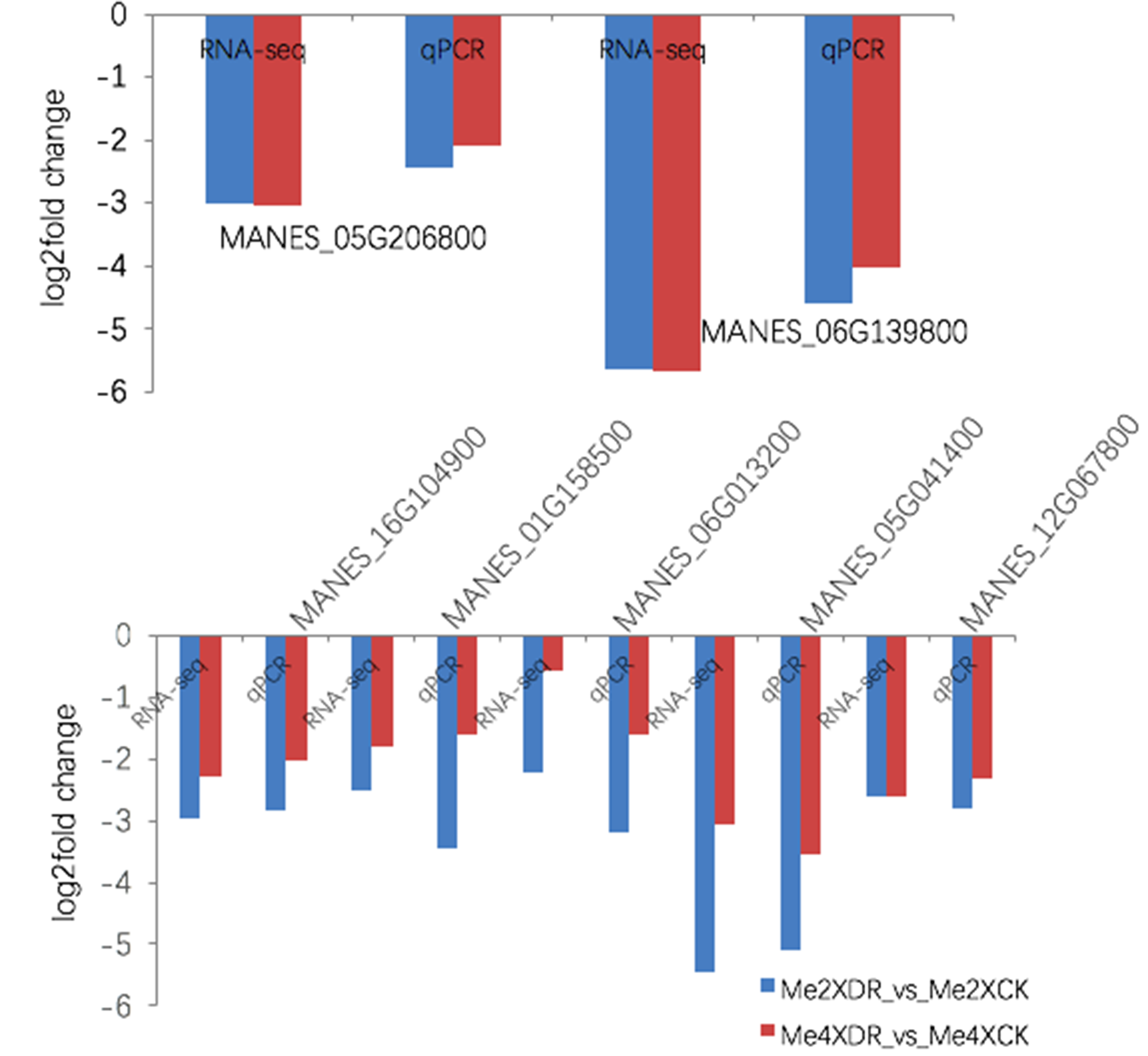

Supplement: Supplementary file 10 — Figure S3. Comparison of the expression of seven genes encoding subtilisin-like protease between Me2XDR_vs._Me2XCK and Me4XDR_vs._Me4XCK detected by qPCR. Bars represent means ± SD of three biological replicates. Cassava β-actin was used as an internal control. (TIF 16466 kb) [file 12864_2019_5895_MOESM10_ESM.tif]
